# Evidence for different molecular parameters in head and neck squamous cell carcinoma of nonsmokers and nondrinkers: Systematic review and meta‐analysis on HPV, p16, and *TP53*

**DOI:** 10.1002/hed.26513

**Published:** 2020-10-23

**Authors:** Frans J. Mulder, Damiana D. C. G. Pierssens, Laura W. J. Baijens, Bernd Kremer, Ernst‐Jan M. Speel

**Affiliations:** ^1^ Department of Otorhinolaryngology and Head & Neck Surgery, GROW‐school for Oncology and Developmental Biology Maastricht University Medical Center Maastricht Netherlands; ^2^ Department of Oral and Cranio‐Maxillofacial Surgery, GROW‐school for Oncology and Developmental Biology Maastricht University Medical Center Maastricht Netherlands; ^3^ Department of Pathology, GROW‐school for Oncology and Developmental Biology Maastricht University Medical Center Maastricht Netherlands

**Keywords:** head and neck cancer, human papillomavirus, nonsmokers, p16, TP53

## Abstract

**Background:**

The goal of this review was to present an overview of the currently identified molecular parameters in head and neck squamous cell carcinoma (HNSCC) of nonsmokers and nondrinkers (NSND).

**Methods:**

Following the PRISMA guidelines, a systematic search was performed using the electronic databases PubMed, Embase, and Google Scholar.

**Results:**

Of the 902 analyzed unique studies, 74 were included in a quantitative synthesis and 24 in a meta‐analysis. Human papillomavirus (HPV) was reported as a molecular parameter in 38 studies, followed by p16 and *TP53* (23 and 14 studies, respectively). The variety of other molecular parameters concerned sporadic findings in small numbers of NSND.

**Conclusions:**

HNSCC in NSND is more often related to HPV and p16 overexpression compared to tumors of smokers‐drinkers. In a third of virus‐negative tumors, *TP53* mutations were detected with a mutational profile associated with aging and ultraviolet light exposure rather than to tobacco consumption.

## 1 BACKGROUND

Head and neck squamous cell carcinoma (HNSCC) usually results from excessive tobacco and alcohol consumption.^1^ A third risk factor in head and neck carcinogenesis is high‐risk human papillomavirus (HPV), especially in the oropharynx.^2^, ^3^ Patients with HPV‐positive oropharyngeal squamous cell carcinoma (OPSCC) usually have a healthier lifestyle without excessive consumption of tobacco and alcohol compared to patients with HPV‐negative tumors.^4^ Additionally, there are HNSCC patients without any exposure to tobacco and alcohol. These nonsmokers and nondrinkers (NSND) appear to be clinically different from their smoking and drinking counterparts: predominantly females at the extremes of age with an early tumor stage, mainly in the oral cavity.^5^, ^6^, ^7^, ^8^, ^9^, ^10^, ^11^ Although these clinical differences have been identified, it is partially unclear what starts the carcinogenesis in this group.

In the past decades, the prevalence rate of HPV in HNSCC has been rising in the United States and Europe and many studies have shown that HPV status is a strong, independent prognostic factor for disease free and overall survival in OPSCC.^12^, ^13^, ^14^ Recently, this has led to a down staging of HPV‐positive OPSCC in the Eighth Edition of the American Joint Committee on Cancer and Union for International Cancer Control tumor‐node‐metastasis classification.^15^, ^16^ An association between HPV positivity and NSND has been suggested in several studies.^17^, ^18^, ^19^

Research into the molecular landscape of HNSCC has increased rapidly in recent years, mainly focusing on differences between these HPV‐positive and HPV‐negative tumors.^3^, ^20^ In addition to new insights into head and neck carcinogenesis, including its intrinsically immunosuppressive nature, this research has revealed other prognostic biomarkers, diagnostic biomarkers, and targets for novel therapeutic options.^3^, ^20^, ^21^, ^22^, ^23^ In this field of molecular research, however, little attention has been paid to processes underlying carcinogenesis in NSND. In this systematic review, an overview of the molecular parameters reported in HNSCC of NSND is presented, including a meta‐analysis on the prevalence of HPV, p16 overexpression, and *TP53* mutations in NSND vs smokers and drinkers (SD).

## 2 METHODS

### 2.1 Search strategy

This systematic review was conducted following the Preferred Reporting Items for Systematic Reviews and Meta‐Analyses.^24^ A systematic search strategy was developed using the electronic databases PubMed, Embase, and Google Scholar combining terms for (a) the head and neck region, (b) squamous cell carcinoma, (c) molecular parameters underlying carcinogenesis, and (d) NSND (Supplementary Table 1). The entire search was performed on October 9, 2018.

### 2.2 Screening

After discarding duplicate articles using EndNote X7.5 (Clarivate Analytics, Philadelphia, Pennsylvania), two independent reviewers (FM, DP) made the first preselecting cut by screening all articles on title and abstract. Inclusion criteria were as follows: (a) original studies on a (b) viral, protein, or genomic parameter (c) in HNSCC, with (d) results on nonsmokers and/or nondrinkers explicitly reported in the title or abstract, (e) published after 1990. Exclusion criteria were as follows: (a) studies in languages other than English, Dutch, or German, (b) data based on animal samples, (c) skin tumors or rare histological variants of HNSCC, and (d) the gray literature >2 years old. After the first selection, the remaining full‐text articles were assessed for eligibility based on the same criteria. Reference lists of included studies and recent systematic reviews on biomarkers in HNSCC were screened for additional literature.^25^, ^26^ If an article was not electronically available, the authors were contacted to obtain the full‐text.

### 2.3 Data extraction and assessment of study quality

For relevant articles, the name of the first author, year of publication, country of conducted research, name of the molecular parameter, tumor location, number of NSND, definition of NSND, study design and method, definition of molecular parameter positivity, and study remarks on the NSND population were retrieved. When at least five articles described the same molecular parameter in NSND, the two reviewers assessed them on methodological quality using a modified 10‐item critical appraisal tool derived from the REporting recommendations for tumor MARKer prognostic studies (REMARK).^27^ The critical appraisal criteria were scored with “yes,” “unclear,” or “no” (Supplementary Table 2). External validity was rated with items 1 to 3, and internal validity with items 4 to 10. Dissonance between the two reviewers was dissolved by discussion.

Data were pooled in a meta‐analysis when (a) a clear and acceptable cutoff value for molecular parameter positivity was reported (as was assessed with items 5, 8, and 10 of the quality assessment), and (b) the number of patients positive and negative for the molecular parameter in both NSND and SD was explicitly reported. Studies reporting that these molecular parameters play no role in the head and neck carcinogenesis of NSND were also included to limit selection and publication bias.

### 2.4 Statistical analysis

Interobserver agreement between the two reviewers for title and abstract screening and full‐text evaluation was determined using Cohen's Kappa coefficient (*к*). For the meta‐analysis, Review Manager 5.3 (The Nordic Cochrane Center, The Cochrane Collaboration, Copenhagen) was used to create forest plots by pooling weighted data, calculating the odds ratio (OR) and 95% confidence intervals (CIs) for a fixed effect of molecular parameter presence in NSND using the Mantel‐Haenszel test. To evaluate the statistical reliability of the data, a sensitivity analysis was performed by only retaining studies in the meta‐analysis with at least 10 patients in both the nonsmokers/nondrinkers and smokers/drinkers groups. In case this did not change the outcome, the smaller studies remained included in the meta‐analysis. The *I* ^2^ statistic was used for heterogeneity estimation of OR variance between studies. Higgins and colleagues proposed adjectives of low, moderate, and high heterogeneity for *I* ^2^ values of 25%, 50%, and 75%, respectively.^28^ Since tumor protein p16 overexpression is a surrogate marker for the HPV status in OPSCC, but not in nonoropharyngeal HNSCC (non‐OPSCC), the presence of HPV and p16 overexpression were analyzed separately for OPSCC and non‐OPSCC.

## 3 RESULTS

### 3.1 Screening and data extraction

**FIGURE 1:**
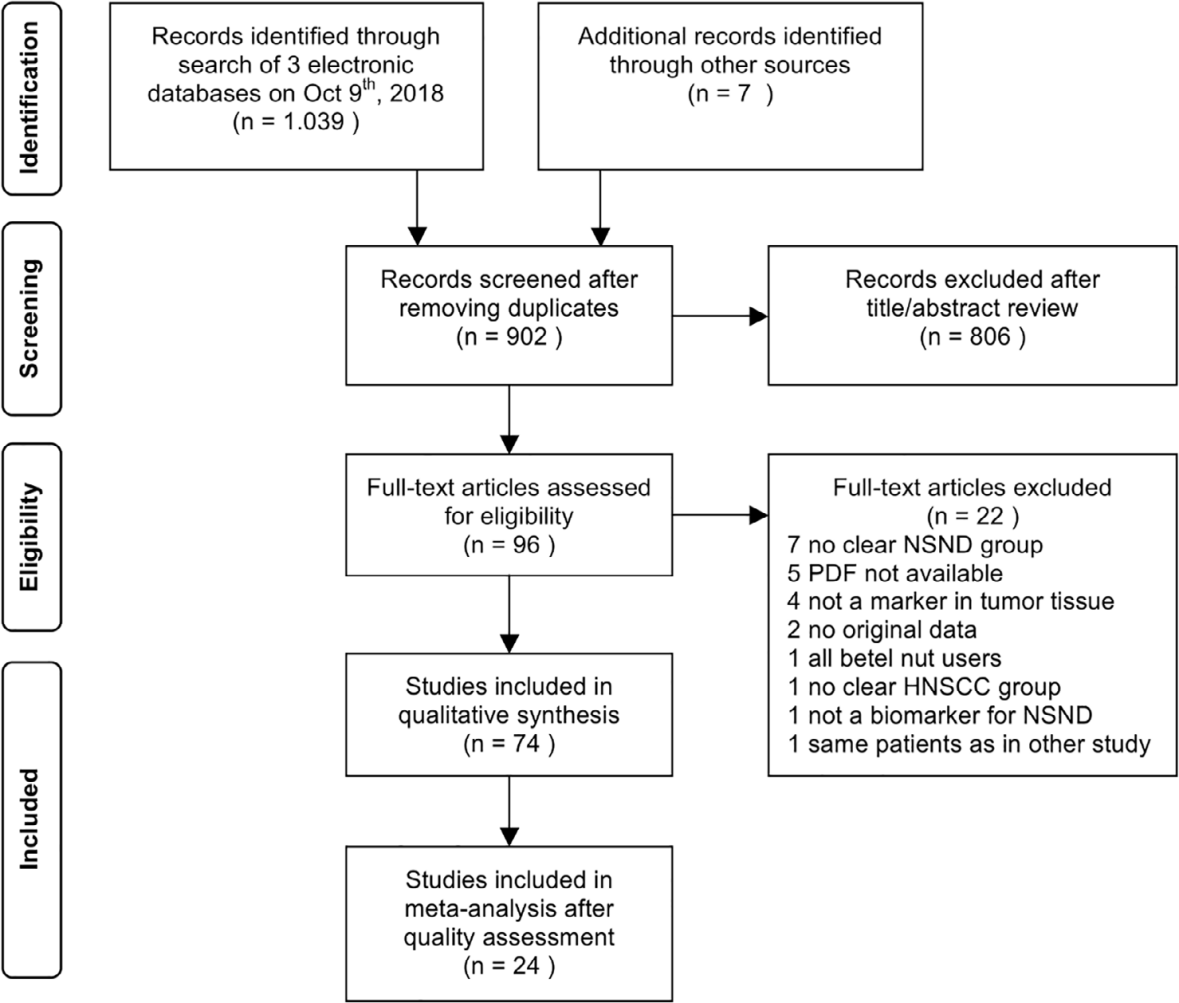
PRISMA flowchart of the literature search. HNSCC, head and neck squamous cell carcinoma; NSND, nonsmokers and nondrinkers

A total of 1039 articles were identified through the electronic search and 7 additional studies from reference lists. After removing duplicates, 902 studies remained for title and abstract evaluation by the two reviewers (*к* = 0.90 for title and abstract inclusion), 96 of which the full texts were read (*к* = 0.88 for full‐text inclusion). Seventy‐four studies were included in the qualitative synthesis (Figure 1).

**FIGURE 2:**
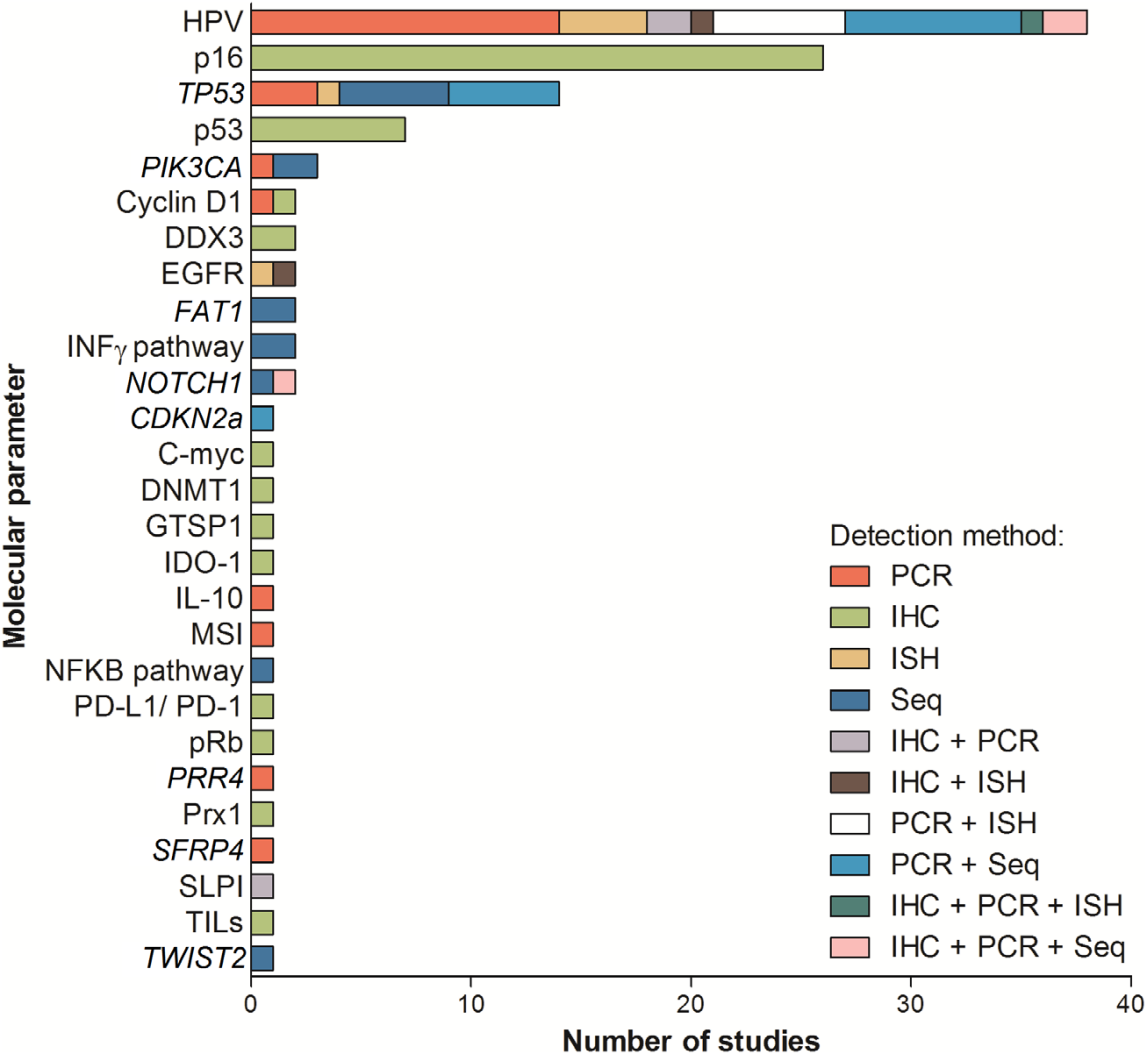
Number of studies and type of detection method of molecular parameter evaluation in head and neck squamous cell carcinoma of nonsmokers and nondrinkers. IHC, immunohistochemistry; ISH, in situ hybridization; PCR, polymerase chain reaction; Seq, sequencing; TILs, tumor‐infiltrating lymphocytes [Color figure can be viewed at wileyonlinelibrary.com]

Most studies were published between 2014 and 2018 (58%; 43/74), with the oldest included study being published in 1991.^29^ Thirty‐nine percent (29/74) of the publications originated from European institutions, 27% (20/74) from North America, 22% (16/74) from Asia, 8% (6/74) from Central‐South America, and 4% (3/74) from Australia. Half of the included studies (38/74) reported on HPV in nonsmokers and/or nondrinkers, in OPSCC (33%) as well as most other subsites of HNSCC: the oral cavity, hypopharynx, and larynx. Two out of the six studies looking specifically at oral tongue squamous cell carcinoma (OTSCC) found HPV DNA in these tumors using polymerase chain reaction (PCR), and one of these two studies also used real‐time nucleic acid sequence‐based amplification.^30^, ^31^ The second most frequently evaluated molecular parameter was tumor protein p16, often used as a surrogate marker for HPV infection. *TP53* mutations, usually present in OTSCC, and p53 protein expression were analyzed in 19% and 10% of the included studies, respectively (Table 1). Although a variety of other molecular parameters have been reported, these concerned sporadic findings and were mostly identified in small numbers of NSND (Figure 2). However, most noticeable were the number of studies indicating a higher impact of the immune response in tumors of NSND compared to SD, with the description of the interferon γ (INFγ) and nuclear factor kappa‐light‐chain‐enhancer of activated B cells (NFKB) pathways, including interleukin‐10 (IL‐10), programmed death‐1 (PD‐1), programmed death‐ligand 1 (PD‐L1), indoleamine 2,3‐dioxygenase 1 (IDO‐1), and tumor‐infiltrating lymphocytes (TILs) (Supplementary Table 3).

**TABLE 1:** HPV, p16, p53, and *TP53* in head and neck squamous cell carcinoma of nonsmokers and nondrinkers

Reference (country)	Molecular parameter	Tumor location	Number of patients (positive cases)		Definition NSND	Study remarks
			NS	ND		
**HPV**						
Amsbaugh et al^32^ (United States)	HPV combined with p16	Oropharynx	79 (NA)	—	NS = never smoking	The rates of HPV/p16 positivity, never smoking, and cervical lymph node metastases were significantly higher for patients with OPSCC of the tonsil, base of tongue, or vallecula subsites when compared with pharyngeal wall or palate subsites
Andrews et al^17^ (United States)	HPV	Oropharynx	18 (14)	18 (14)	NSND = no prior or current use of tobacco and/or alcohol	HR‐HPV infection is a predominant risk factor in the development of OPSCC in patients who do not smoke or drink
Ang et al^33^ (United States)	HPV	Oropharynx	73 (59)	—	NS = never smoked	HPV‐positive oropharyngeal cancer was more common among patients who had never smoked
Angiero et al^34^ (Italy)	HPV	Oral cavity	11 (3)	11 (3)	NSND = nonsmoker and nondrinker patients	The presence of HPV DNA appeared to be a molecular marker in dysplasia and OSCC of a subgroup of nonsmoker and nondrinker patients
Antonsson et al^35^ (Australia)	HPV	Head and neck Oropharynx	19 (0) 8 (7)	20 (2) 6 (5)	NSND = self‐reported lifelong nonsmoker, nondrinker	HPV prevalence and p16 overexpression were highest in OPSCC, younger patients, and nonsmokers
Bragelmann et al^36^ (United States)	Viral mRNA	Oral tongue	7 (0)	7 (0)	NS < 5PY ND ≤ 1 glass of wine or equivalent/day	None of the seven OTSCC showed significant presence of viral transcripts
Chen et al^37^ (China)	HPV	Oral cavity	89 (NA)	105 (NA)	NS < 100 cig/lifetime ND < 1 drink/week for at least 6 months	Oral HPV infection was strongly associated with an increased risk of OSCC in females, young adults, married population, merchants, nonsmokers, nonalcohol drinkers, and nontea drinkers
Chen et al^38^ (Taiwan)	HPV	Larynx	13 (4)	55 (10)	—	Patients with HPV‐positive tumors were older, less local/regional recurrence, and nonsmoker. A low prevalence of HPV infection in our series suggests that HPV is not a major cause of LSCC
Chen et al^39^ (China)	HPV	Larynx	70 (13)	110 (12)	NS = never smoking ND = never drinking	The risk of LSCC associated with HPV‐16 DNA positivity was even higher in patients aged 55 years or younger, males, never smokers, and never drinkers
Chuang et al^40^ (China)	HPV	Oral cavity	73 (53)	99 (64)	—	HPV‐16/18 infection rates in females, nonsmokers, nondrinkers, and nonbetel quid chewers were higher than in males, smokers, drinkers, and betel quid chewers
Dediol et al^41^ (Croatia)	HPV	Oral cavity	77 (17)	77 (17)	NS < 10PY ND = no alcohol on daily basis	In contrast to OPSCC, HPV in OSCC is a negative predictive factors [for disease‐specific survival], especially in NSND patients
Descamps et al^42^ (Belgium)	HPV	Head and neck	24 (6)	50 (12)	NSND = never used tobacco or alcohol	We observed a significantly worse prognosis for consumers of alcohol and tobacco compared to nondrinkers and nonsmokers
Farnebo et al^43^ (Sweden)	HPV	Head and neck	26 (20)	—	NS = never smoker	HPV‐positive never smokers had lower frequencies of *TP53* mutations.
Farshadpour et al^18^ (Netherlands)	HPV combined with p16	Oropharynx	16 (12)	16 (12)	NSND = no history of smoking tobacco and alcohol consumption	All HPV‐positive tumors showed p16 overexpression. HPV is strongly associated with OPSCC of nonsmoking and nondrinking patients
Fouret et al^44^ (France)	HPV	Head and neck	10 (5)	—	NS = 0PY	HPV may play a role in HNSCC in nonsmokers
Gillison et al^45^ (United States)	HPV	Head and neck	23 (16)	23 (16)	NS < 1 cig/day for a year ND < 1 alcoholic drink/day for a year	Compared with subjects who neither smoked tobacco nor drank alcohol, those with heavy use of tobacco and alcohol had an increased risk of HPV‐16‐negative HNSCC
Gonzalez‐Ramirez et al^46^ (Mexico)	HPV	Oral cavity	42 (4)	47 (4)	NSND = no current or former tobacco or alcohol use	All HR‐HPV‐positive OSCC cases corresponded to young patients, nonsmokers, and nonalcohol drinkers
Hafkamp et al^47^ (Netherlands)	HPV	Oropharynx	12 (10)	31 (18)	NS = never smoker or former smoker >10 years before SCC ND ≤2 whiskey equivalents/day	The presence of HPV‐16 proved to be a strong independent predictor of favorable outcome in nonsmokers
Hoffmann et al^48^ (Germany)	HPV combined with p16	Oropharynx	36 (NA)	—	NS ≤10PY	Nonsmoking HPV‐positive TSCC patients show 10‐year OS of 100% and 90.9% PFS when treated with adjuvant RCT
Hong et al^49^ (Australia)	HPV combined with p16	Oropharynx	73 (59)	53 (32)	NS = nonsmoker ND = nondrinker	Our data show a rising prevalence of HPV‐positive OPSCC in Australia over the last two decades, with patients presenting at an older age and about one third have never smoked
Joo et al^50^ (Korea)	HPV	Hypopharynx	18 (5)	28 (5)	NS = never smoked ND = nondrinkers	Significant correlations were found between positive HR‐HPV and younger age and nonsmoking status
Laco et al^19^ (Czech Republic)	HPV	Oral cavity Oropharynx	24 (3) 22 (18)	24 (3) 22 (18)	NSND = no history of either smoking or chronic alcohol abuse	The majority of tumors developing in patients with OPSCC without positive personal history of smoking and alcohol abuse are related to oral HPV infection, whereas the viral etiology is responsible for a substantially smaller subset of OSCC
Li et al^51^ (United States)	HPV	Oral tongue	6 (0)	—	NS = no history of tobacco smoking or chewing	No HPV was found in any of the tumors other than the HPV‐positive control
Maruyama et al^52^ (Japan)	HPV	Oropharynx	22 (13)	37 (20)	NS ≤5PY ND < 5 units of sake/day for a year	In OPSCC, which showed an increasing trend of HPV prevalence over time, HPV infection was inversely correlated with tobacco smoking, alcohol drinking, *TP53* mutations, and a disruptive [gene] mutation
Mena et al^53^ (Spain)	HPV combined with p16	Oropharynx	82 (29)	137 (32)	NS = nonsmoker ND = nondrinker	Being non‐smoker or nondrinker was consistently associated across HPV‐relatedness definitions with HPV positivity
Oliveira et al^31^ (Brazil)	HPV	Oral cavity	16 (7)	—	NS = never smoked	The tongue was the most prevalent infected anatomical site. A significant number of HPV samples were positive among nonsmoking patients
Peterson et al^54^ (United States)	HPV	Head and neck	96 (52)	67 (NA)	NS = self‐reported never smoker	In HPV‐positive patients, for overall, recurrence‐free, and disease‐specific survival, nonsmokers showed marginal improvements in survival compared to smokers
Platek et al^55^ (United States)	HPV	Oropharynx	26 (22)	—	NS = never smoker	When HPV status was stratified by smoking status, the OS favored never/former smokers vs current smokers, but the difference only reached statistical significance for patients with HPV‐positive tumors
Poling et al^56^ (United States)	HPV	Oral tongue	44 (0)	57 (0)	NS = never tobacco on regular basis ND < 10 units/week	HPV E6/E7 mRNA transcripts were detected in only 1 [smoker] case
Quabius et al^57^ (Germany)	HPV	Head and neck	60 (31)	—	NS = nonsmoker	The surplus of annexin A2 in nonsmokers and HPV‐positive patients supports our hypothesis that decreased SLPI levels facilitate HPV infection
Schlecht et al^58^ (United States)	HPV	Head and neck	7 (3)	21 (7)	NS = nonsmoker ND = light drinker <4 drinks/week for 3 years	Focusing on never smokers, we identified a distinct subset of 123 genes that were specifically dysregulated in HPV16‐positive HNSCC
Siebers et al^59^ (Netherlands)	HPV	Oral tongue	7 (0)	7 (0)	NS = never smoked ND ≤1 unit alcohol/day	No HPV was detected in these specimens.
Simonato et al^60^ (Brazil)	HPV	Oral cavity	3 (2)	11 (3)	NS = no tobacco consumption ND = no alcohol consumption	The highest prevalence of HPV DNA was observed in nonsmoking patients over the age of 60 years.
Tachezy et al^61^ (Czech Republic)	HPV	Oral cavity/ oropharynx	7 (7)	16 (11)	NS = smoked <0.5 pack/week for a year ND < 1 drink/week for a year	The prevalence of HPV DNA was lower in OSCC than in OPSCC, and higher in NSND.
Tsimplaki et al^30^ (Greece)	HPV	Oral tongue	15 (5)	15 (5)	NSND = no tobacco and no alcohol use	HPV infection was strongly associated with abstinence from tobacco and alcohol.
Vatca et al^62^ (United States)	HPV	Oropharynx	42 (38)	—	NS = stopped >1 year before diagnosis and < 10 PY	Risk factors for OPSCC modify the incidence of treatment‐related early toxicities, with HPV‐positive and nonsmoking status correlating with increased risk of high‐grade mucositis
Wangsa et al^63^ (United States)	HPV	Oral tongue	20 (0)	—	NS = not smoking	The one patient that tested positive for HPV‐16 was a Stage 4 patient that smoked
Xu et al^64^ (China)	HPV combined with p16	Larynx	115 (12)	236 (17)	NS = smoking < once/week ≥1 year ND < 1 unit alcohol/week ≥1 year	HPV infection was more common among nonsmokers, nondrinkers, and patients with supraglottic LSCC.
**p16**						
Angiero et al^34^ (Italy)	p16	Oral cavity	11 (6)	11 (6)	—	No specific remark regarding p16 and nonsmokers and/or nondrinkers
Antonsson et al^35^ (Australia)	p16	Head & neck	26 (8)	24 (8)	NSND = self‐reported lifelong nonsmoker, nondrinker	p16 overexpression was highest in OPSCC, younger patients, and nonsmokers.
Dediol et al^41^ (Croatia)	p16	Oral cavity	77 (21)	77 (21)	—	In contrast to OPSCC, p16 expression in OSCC is a negative predictive factor [for disease specific survival], especially in NSND patients.
Gillison et al^65^ (United States)	p16	Oropharynx	96 (81)	—	NS = never smoker	p16 positive patients were more likely to be never smokers and had significantly lower cigarette smoking exposure.
Haas et al^66^ (Germany)	p16	Oropharynx	24 (7)	24 (7)	NS < 10PY ND = no alcohol on daily basis	p16 was the only marker showing a significant correlation with a negative smoking history.
Habbous et al^4^ (Canada)	p16	Oropharynx	755 (NA)	2032 (NA)	NS = never smoked ND = no/light alcohol consumption (≤ 2 drinks/day)	Variables associated with p16‐positive status are male sex, tonsillar or base‐of‐tongue tumors, smaller tumors, nodal involvement, less smoking and lower alcohol consumption.
Heaton et al^67^ (United States)	p16	Oral tongue	50 (5)	—	NS < 100 cig in lifetime	There was no correlation found between p53 and p16 IHC status and the clinicopathologic variables studied.
Hess et al^68^ (United States)	p16	Oropharynx	66 (60)	30 (24)	NS = never smokers ND = self‐reported rare alcohol use	Self‐reported heavy alcohol use was significantly higher among p16‐negative patients and more p16‐positive patients identified themselves as “never smokers”
Kalfert et al^69^ (Czech Republic)	p16	Larynx	8 (6)	—	NS = nonsmoker	p16 expression in glottic LSCC, especially in subgroup of nonsmokers, might be a promising prognosticator of better clinical outcome in routine practice.
Karpathiou et al^70^ (France)	p16	Head & neck	6 (4)	—	NS = not smoking	p16 positivity and p53 normal expression were significantly correlated with nonsmoking, an earlier T stage and a nonkeratinizing morphology.
Laco et al^19^ (Czech Republic)	p16	Oral cavity Oropharynx	24 (7) 22 (22)	24 (7) 22 (22)	NSND = no history of either smoking or chronic alcohol abuse	In this population of NSND, p16 expression was detected in 29% of OSCC and 100% of OPSCC
Mafune et al^71^ (Japan)	p16	Head & neck	35 (2)	—	NS ≤10PY prior to surgery or stopped ≥20 years ND < 1 drink/day	Nonsmokers did not differ significantly from smokers with regard to p16
Poling et al^56^ (United States)	p16	Oral tongue	44 (5)	57 (5)	NS = never tobacco on regular basis ND < 10 units/week	p16 overexpression was detected in 9 of 78 cases
Ralli et al^72^ (India)	p16	Head & neck	10 (7)	29 (21)	—	Expression of p16 was higher in nonsmokers and nonalcohol consumers and significantly associated with paan chewing habit.
Silva et al^73^ (Brazil)	p16	Oropharynx/ larynx	4 (4)	13 (7)	NS = no smoking habit ND = no alcohol consumption	p16 expression was more intense in nonsmoking patients, whose tumors showed negative vascular embolization, negative lymphatic permeation, and clear surgical margins.
Ye et al^74^ (Canada)	p16	Oropharynx	52 (45)	—	NS = never smoker	Most patients were p16‐positive, were younger (predominantly male), mostly former or nonsmokers, and had a more advanced nodal stage.
Zhao et al^75^ (United States)	p16	Oropharynx	5 (2)	—	NSND = never smoker never drinker	Different p16 protein localization suggested different survival outcomes in a manner that does not require limiting the biomarker to the oropharynx and does not require assessment of smoking status
**p53**						
Angiero et al^34^ (Italy)	p53	Oral cavity	11 (9)	11 (9)	—	No specific remark regarding p53 and nonsmokers and/or nondrinkers
Fernandez‐Acenero et al^76^ (Spain)	p53	Larynx	21 (8)	21 (8)	NSND = never smoked or drank alcohol	p53 expression seems to negatively influence survival in nonsmoking nonalcoholic patients with LSCC.
Field et al^29^ (United Kingdom)	p53	Head & neck	7 (1)	—	NS = nonsmoker	Six out of seven nonsmokers did not express p53 whereas 29 of 37 heavy smokers were found to have elevated p53 expression
Haas et al^66^ (Germany)	p53	Head & neck	24 (17)	24 (17)	NSND = never used tobacco or alcohol on a regular basis	Expression of p53 was independent of smoking history and tumor site
Heaton et al^67^ (United States)	p53	Oral tongue	51 (16)	—	NS < 100 cig in lifetime	There was no correlation found between p53 and p16 IHC status and the clinicopathologic variables studied
Karpathiou et al^70^ (France)	p53	Head & neck	6 (3)	—	NS = not smoking	p16 positivity and p53 normal expression were significantly correlated with nonsmoking, an earlier T stage and a nonkeratinizing morphology
Matthews et al^77^ (Netherlands)	p53	Oral tongue	14 (7)	9 (6)	NSND = nonsmokers nondrinkers	There was an apparent negative association between IHC detection of p53 and tobacco smoking and/or alcohol intake
***TP53***						
Faden et al^78^ (United States)	*TP53*	Oral tongue	43 (NA)	—	NS = never smoker	OTSCC in nonsmokers have *TP53* mutation rates similar to other HNSCC, yet these mutations do not appear related to carcinogen exposure based on the mutational spectrum and clinical history
Farnebo et al^43^ (Sweden)	*TP53*	Head & neck	7 (3)	—	NS = never smoker	HPV‐positive never smokers had lower frequencies of *TP53* mutation
Fouret et al^44^ (France)	*TP53*	Head & neck	10 (0)	0	NS = 0PY	There were no *TP53* gene mutations in cancer cells
Heaton et al^67^ (United States)	*TP53*	Oral tongue	47 (10)	—	NS < 100 cig in lifetime	*TP53* and *CDKN2a* mutations in never‐smoker OTSCC are associated with worse clinicopathologic characteristics and poorer survival outcomes
Hong et al^79^ (Australia)	*TP53*	Oropharynx	33 (10)	38 (14)	NS = never smoker ND = never drinker	Among patients with HPV‐positive OPSCC, there was no significant difference in *TP53* mutation by smoking status. HPV‐positive OPSCC are less likely to have mutant *TP53* than HPV‐negative OPSCC
Li et al^51^ (United States)	Gene mutations, including *TP53*	Oral tongue	6 (1)	—	NS = no history of tobacco smoking or chewing	The recurrently mutated genes in our cohort of cancers from nonsmokers were *CTNNA3*, *EIF3A*, *EP300*, *FXR1*, *NEK8*, *NOTCH1*, *PIK3CA*, *PKHD1L1*, *PTCHD2*, *RALGAPB*, *SPEN*, and *UBR4*. Nonsmokers had fewer *TP53* mutations than smokers
Mafune et al^71^ (Japan)	*TP53*	Head and neck	71 (37)	89 (50)	NS ≤10PY prior to surgery or stopped ≥20 years ND < 1 drink/day	In nonsmokers, 24% of *TP53* mutations occurred at CpG sites, but in smokers, 12% did
Maruyama et al^52^ (Japan)	*TP53*	Head & neck	47 (15)	59 (14)	NS ≤5PY ND < 5 units of sake/day for a year	In OPSCC, HPV infection was inversely correlated with tobacco smoking, alcohol drinking, *TP53* mutations, and a disruptive [gene] mutation
Mirghani et al^80^ (France)	Mutation profiles, including *TP53*	Oropharynx	37 (3)	—	NS = without any smoking history	Mutation rate [of *TP53*] was not significantly different in smokers compared with nonsmokers, even when analyses focused on heavy smokers
Ostwald et al^81^ (Germany)	*TP53*	Oral cavity	23 (10)	26 (9)	NSND = no history of smoking or drinking	The rate of lip tumors with mutations was higher in nonsmokers than in smokers. In contrast, *TP53* mutations in intraoral tumors clustered in smokers
Pickering et al^82^ (United States)	Gene mutation frequencies, including *TP53*	Oral tongue	44 (31)	—	NS < 1PY smoking history	Three genes showed trends toward statistical significance: *FAT1*, *TP53*, and *PIK3CA*. However, not between the younger and older patient cohorts
Tan et al^83^ (Singapore)	Mutation profiles, including *TP53*	Oral tongue	25 (3)	—	NS = never smoker	There was no significant association between smoking history and the presence of any mutation detected by the LungCarta panel, or specific alterations in *MET*, *TP53*, and *STK11*
Wangsa et al^63^ (United States)	FISH markers, including *TP53*	Oral tongue	20 (NA)	—	NS = not smoking	Copy number increases of all five markers were found to be correlated to nonsmoking habits, while smokers in this cohort had low‐level copy number gains
Zanaruddin et al^84^ (Malaysia)	*TP53*	Oral cavity	24 (8)	24 (8)	NSND = no smoking, alcohol or chewing habit	*TP53* truncating mutations were more common in patients with no risk habits

Abbreviations: cig, cigarettes; HNSCC, head and neck squamous cell carcinoma; HPV, human papilloma virus; HR, high risk; IHC, immunohistochemistry; LSCC, laryngeal squamous cell carcinoma; NA, not available; ND, nondrinkers; NS, nonsmokers; OPSCC, oropharyngeal squamous cell carcinoma; OS, overall survival; OSCC, oral squamous cell carcinoma; OTSCC, oral tongue squamous cell carcinoma; PFS, progression‐free survival; PY, pack‐years; RCT, radiochemotherapy; SCC, squamous cell carcinoma; TSCC, tonsillar squamous cell carcinoma.

### 3.2 Assessment of study quality

Eleven included studies met all criteria for external validity,^37^, ^41^, ^44^, ^45^, ^54^, ^59^, ^61^, ^62^, ^64^, ^67^, ^71^ whereas another study met all criteria for internal validity.^35^ Five out of seven criteria for internal validity were met in 10 studies.^18^, ^53^, ^58^, ^59^, ^68^, ^73^, ^75^, ^79^, ^80^, ^84^ Whether or not the molecular parameter was interpreted without knowledge of the patients' clinical characteristics was the most frequently underreported critical appraisal item (20% scored yes), followed by the items univariate and multivariable statistics in particular for NSND (32%) and the item of a clear NSND definition (34%)(Supplementary Table 4).

A molecular parameter in an exclusively NSND population was reported in 14 studies.^17^, ^18^, ^19^, ^30^, ^34^, ^36^, ^41^, ^45^, ^59^, ^76^, ^84^, ^85^, ^86^, ^87^ For the other studies, it was unclear if the nonsmokers were the same patients as the nondrinkers and vice versa. There was a large variety in definitions for considering someone as a NSND. Usually, it was a general definition like “never used tobacco or alcohol.” More specific definitions for nonsmoking varied from “<100 cigarettes in their lifetime” to “…smoked less than 10 pack‐years prior to the surgical resection of HNSCC.”^67^, ^71^ For nondrinking, the definitions ranged from never having “consumed at least 1 drink/week continuously for at least 6 months” to “drinking less than five units of sake (=140 g alcohol) per day for 1 year” (Table 1).^37^, ^52^

### 3.3 Meta‐analysis on molecular parameters HPV, p16 overexpression, and TP53 mutations

**FIGURE 3:**
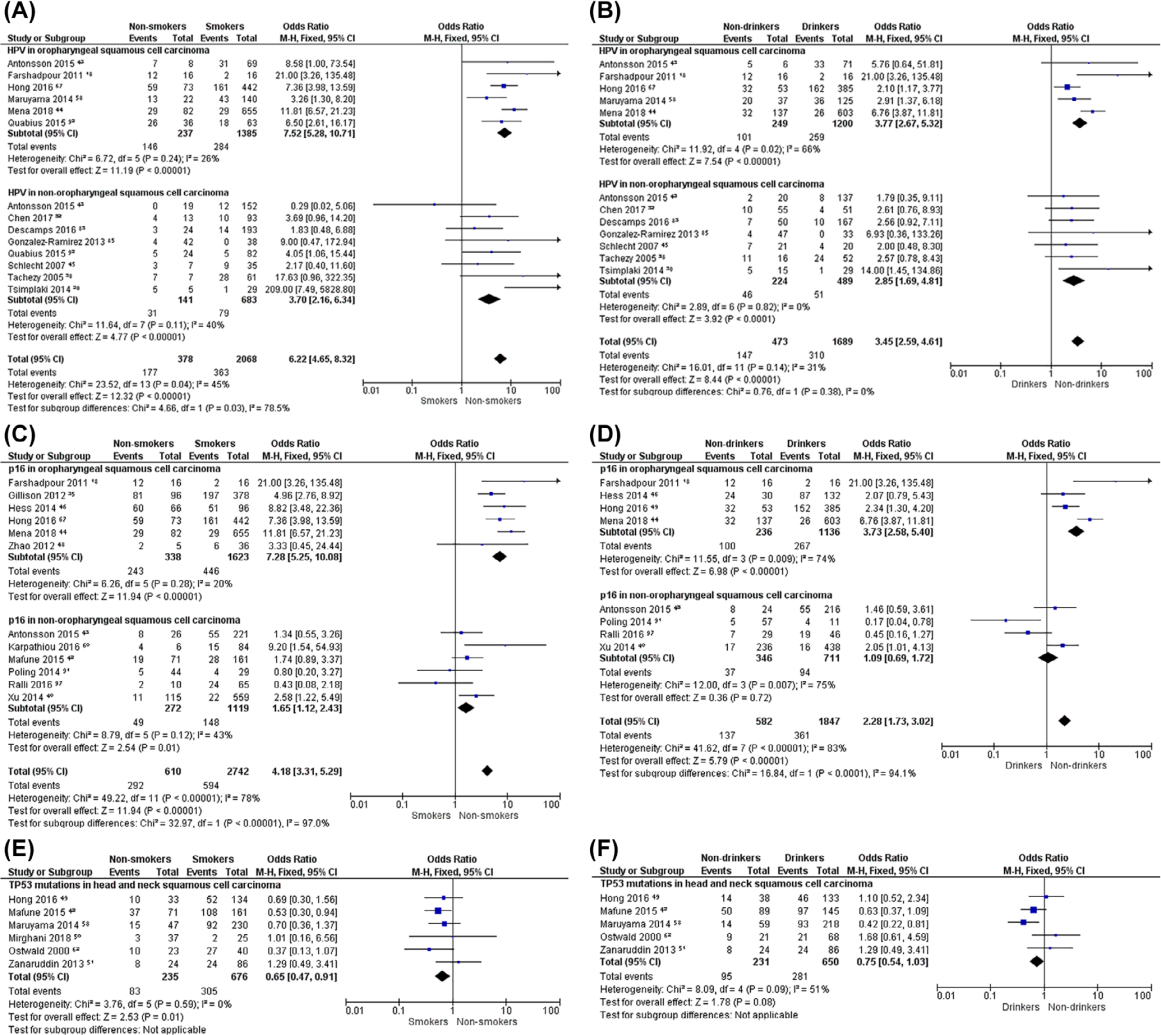
Meta‐analysis on the prevalence of molecular parameters HPV (A,B), p16 overexpression (C,D) and TP53 mutations (E,F) in head and neck squamous cell carcinoma of nonsmokers vs smokers (A,C,E) and nondrinkers vs drinkers (B,D,F). The presence of HPV and p16 overexpression were analyzed separately for oropharyngeal and nonoropharyngeal squamous cell carcinoma [Color figure can be viewed at wileyonlinelibrary.com]

Twelve studies detected HPV presence using at least two identification techniques in HNSCC of nonsmokers, and all but one of these studies reported on nondrinkers too. HPV‐16 was the most frequently detected parameter, followed by HPV‐18 and HPV‐33. Furthermore, HPV types 31, 35, 51, 56, and 58 were described as well, although it was not specified if these types were present in the NSND and/or SD population. HPV was found significantly more frequent in NSND compared to SD (OR_nonsmoker_ = 6.22, 95% CI 4.65‐8.32, *P* < .001, *I* ^2^ = 45%; OR_nondrinker_ = 3.45, 95% CI 2.59‐4.61, *P* < .001, *I* ^2^ = 31%) (Figure 3A,B). This significant difference in prevalence was more pronounced in OPSCC, with a pooled prevalence of 62% (n = 146/237) in nonsmokers and 41% (n = 101/249) in nondrinkers, compared to a HPV prevalence of 21‐22% (n = 284/1.385 and n = 259/1200) in the SD group. In non‐OPSCC, the HPV prevalence was approximately 22% (n = 31/141 and n = 46/224) in NSND vs 11% (n = 79/683 and n = 51/489) in SD.

Of the 12 studies describing a strong and diffuse p16‐staining pattern in tumors of nonsmokers, 8 presented data on nondrinkers as well. In OPSCC, p16 overexpression was significantly more prevalent in nonsmokers (OR = 7.28, 95% CI 5.25‐10.08, *P* < .001, *I* ^2^ = 20%) and nondrinkers (OR = 3.73, 95% CI 2.58‐5.40, *P* < .001, *I* ^2^ = 74%) compared to SD. Similar results were found in non‐OPSCC of nonsmokers vs smokers (OR = 1.65, 95% CI 1.12‐2.43, *P* = .01), which just remained significantly different after sensitivity analysis (OR = 1.51, 95% CI 1.01‐2.26, *P* = .04) (Supplementary Figure 1), but not in nondrinkers vs drinkers (OR = 1.09, 95% CI 0.69‐1.72, *P* = .72) (Figure 3C,D).

Tumor protein p53 could not be pooled because definitions for positivity were too heterogeneous, ranging from a “clear brown color, regardless of the staining intensity” and “>5% staining” to “≥50% nuclear/cytoplasmic staining.”^66^, ^67^, ^70^, ^77^ When looking at least at exon 5‐8 (coding the DNA binding portion of p53 and containing >90% of the mutations described in HNSCC), *TP53* mutations were found in 35% of the 235 nonsmokers presented in the six included studies.^54^ Though this percentage is significantly lower than the prevalence of *TP53* mutations found in the smokers group of these studies (45% [n = 305/676], OR = 0.65, 95% CI 0.47‐0.91, *P* = .01), it still is a considerable percentage. When pooling the data on nondrinkers and drinkers, there was no significant difference in *TP53* mutation prevalence (OR = 0.75, 95% CI 0.54‐1.03, *P* = .09), with 41% of the 231 nondrinkers having a *TP53* mutation (Figure 3E,F). The *TP53* mutations usually consisted of a G:C‐A:T transition, which is a mutational signature related to aging and ultraviolet light exposure.^52^, ^81^, ^84^, ^88^ In addition, mutations in the abovementioned studies were reported in exons 4 to 8 and 10, repeatedly at a CpG site, and were less common in nasopharyngeal squamous cell carcinoma and HPV‐positive OPSCC.^52^, ^71^, ^79^, ^80^, ^81^, ^84^

## 4 DISCUSSION

The rapidly developing field of molecular research is identifying a growing number of biomarkers for cancer diagnosis, prognosis, therapy selection, or therapy effect evaluation. Despite the rich body of molecular data on HNSCC in SD, there is little comprehensive information on specific molecular parameters underlying carcinogenesis in NSND, in which the carcinogenesis is expected to be different. In the reviewed literature, the most prevalent and most frequently reported molecular parameters in NSND are well known from tumors in SD: HPV, tumor protein p16 overexpression, *TP53* mutations, and tumor protein p53 immunohistochemistry (IHC). Nonetheless, there is substantial heterogeneity in definitions for both constructs; NSND and parameter positivity. The current meta‐analysis showed a higher prevalence of HPV in both OPSCC and non‐OPSCC of NSND compared to HNSCC in SD. Similar results were found for p16 overexpression in OPSCC of NSND and in non‐OPSCC of nonsmokers. Remarkably, specific *TP53* mutations were detected in more than a third of the included NSND.

A great variety in the definition of the construct NSND was found in the literature, even including descriptions such as “less than 10 pack years prior to the surgical resection of HNSCC” or “drinking <140 g alcohol/day for a year.”^52^, ^71^ The International Head and Neck Cancer Epidemiology (INHANCE) consortium encountered a similar variety in the definition of the construct NSND in their pooled data analysis from patients in Europe and the Americas, with definitions such as “smoking one‐half pack or more per week for ≥1 year” and “consumed an average of one or more drinks per week for 1 or more years” for smokers and drinkers, respectively.^89^ For smoking, an accurate definition seems necessary, as the INHANCE consortium concluded that there is no harmless level of tobacco consumption, with already an increased risk of getting HNSCC when smoking >0‐3 cigarettes/day.^90^ In their re‐analysis of case‐control studies, Dal Maso and colleagues also found a steep increase in HNSCC risk with increased tobacco consumption, starting from 1 cigarette/day, regardless of ethanol intake.^1^ However, for alcohol, there seems to be a threshold effect at approximately 50 g/day in nonsmokers before the increased HNSCC risk starts.^1^ Therefore, when analyzing nondrinkers, a less strict definition of the construct nondrinking may be opted for.

The present meta‐analysis showed that the HPV and p16 overexpression prevalence in OPSCC was over 60% (n_HPV_ = 146/237 and n_p16_ = 243/338) in nonsmokers and 40% (n_HPV_ = 101/249 and n_p16_ = 100/236) in nondrinkers, compared to 20% (n_HPV_ = 284/1.385, n_HPV_ = 259/1.200, n_p16_ = 446/1.623, and n_p16_ = 267/1.136) in SD. A wide range of HPV prevalence has been reported in both OPSCC and non‐OPSCC, summarized by Kreimer and colleagues in their systematic review of 60 studies, with an overall HPV prevalence of 36% (n = 345/969) in OPSCC.^91^ This is higher than the HPV prevalence in SD of the current meta‐analysis, but HPV status was solely based on PCR results and the smoking and drinking habits of the patients were not reported in the study by Kreimer and colleagues. Our results are in concordance with other studies analyzing large cohorts of OPSCC based on HPV DNA in combination with either E6*I mRNA or p16 IHC, where a HPV prevalence of 22% (n = 243/1.085) was found, rising up to 50% to 60% (patient numbers not displayed) in patients from South America, Northern Europe, Central Eastern Europe, and Australia, and going further up to 80% (n = 59/73) in nonsmokers.^49^, ^92^ Although the first phase III de‐escalation trial for HPV‐positive OPSCC had turned out in favor of the standard treatment cisplatin‐based (opposed to cetuximab‐based) chemoradiotherapy, including >50% nonsmokers (defined as “never smoked”) in both study arms, results of other trials are still being awaited.^93^, ^94^ Therefore, the higher HPV prevalence in NSND might affect the treatment strategy of these patients considerably.

The present meta‐analysis determined a HPV prevalence just over 20% (n = 31/141 and n = 46/224) in non‐OPSCC of NSND, being comparable to the HPV prevalence in OPSCC of SD (n = 284/1385 and n = 259/1200). The SD with non‐OPSCC had a significantly lower HPV prevalence of 11% (n = 79/683 and n = 51/489). These percentages are higher than Castellsagué and colleagues found in their analysis of oral (n = 1264) and laryngeal (n = 1042) squamous cell carcinoma, with a HPV prevalence up to 7% in South America, Central America, and Northern Europe.^92^ This difference might be the result of inclusion of more recent studies in the present systematic review in combination with a worldwide rising HPV prevalence, or because of a higher prevalence in NSND. Kreimer and colleagues reported an overall HPV prevalence in non‐OPSCC similar to the prevalence of the NSND.^91^ Again, this might be an overestimation since these data are only based on HPV detection using PCR and on the HPV prevalence including SD.

Contrary to expectations, the p16 overexpression and HPV prevalence were similar, both in OPSCC and non‐OPSCC. Therefore, it has been recommended to combine PCR, ISH, IHC, or sequencing assays for obtaining an optimal sensitivity and specificity for biologically active HPV detection.^53^, ^95^, ^96^ This is clinically relevant because only OPSCC with transcriptionally active HPV are related to a better survival compared to biologically inactive variants.^53^, ^96^ This difference in sensitivity/specificity between HPV DNA and p16 IHC detection was reported in several studies reviewed in the present meta‐analysis too, with none of the studies presenting a perfect relationship between HPV DNA and p16 IHC detection, neither in OPSCC nor in non‐OPSCC.^18^, ^35^, ^49^, ^53^ Therefore, only studies confirming the presence of HPV with at least two techniques were included in this meta‐analysis, with p16 IHC being a valid confirmation technique in OPSCC when there was ≥70% positivity or diffuse intense/strong staining (Supplementary Table 2).

Following genome sequencing data, signatures of *TP53* mutational processes in human cancers have previously been determined.^88^, ^97^ Signatures contributing to a significant number of somatic *TP53* mutations in HNSCC include signature 1B (associated with aging), signature 2 (associated with apolipoprotein B editing complex), signature 4 (associated with smoking), and signature 7 (associated with ultraviolet light exposure). Signature 1 is related to relatively elevated rates of spontaneous deamination of 5‐methyl‐cytosine that are acquired over a human lifetime, at a relatively constant rate in normal somatic tissue that is similar in different people, which may result in cancer in elderly people via C > T transitions.^97^ This mutation is in concordance with the *TP53* G:C‐A:T transitions reported in two of the included studies of this meta‐analysis (C > T in 14% (1/7) and 41% (9/22)).^52^, ^84^ An explanation for a higher prevalence of this signature could be the typically higher age of NSND compared to SD.^7^, ^9^, ^10^, ^11^ Signature 7 shows a higher prevalence of C > T mutations in untranscribed strands of genes following ultraviolet light exposure, impairing the transcription‐coupled nucleotide excision repair. This fits the C > T mutations found in lip tumors of one included study (C > T in 60% [6/10]), where sunlight might play a dominant role in squamous cell carcinoma of the lip area between the vermilion border and wet line.^81^ Although C > A mutations, typical for smoking‐related tumors as a result of the tobacco carcinogen benzo[*α*]pyrene, have previously been observed in smaller numbers of oral cavity and pharyngeal tumors of nonsmokers, this finding could not be confirmed in the current meta‐analysis.^88^ These data strengthen the premise of a different pathway of carcinogenesis resulting in *TP53* mutations in HNSCC of NSND compared to SD, with a more prominent role of spontaneous C > T mutations acquired over a patient's lifetime as a result of aging in the former group, opposed to C > A mutations resulting from tobacco exposure in the latter group.

*TP53* mutations are of interest as a biomarker because tumors containing these are associated with a more aggressive and therapy‐resistant phenotype.^98^, ^99^ Many studies analyzed the concordance between *TP53* mutations and its gene product, p53 protein expression, as a cheaper and faster IHC assay.^100^, ^101^ In addition, p53 activity is often inactivated following the expression of oncoprotein E6.^34^ However, discrepancies have been reported between p53 IHC and the mutational status of the *TP53* gene.^100^, ^101^ Possible explanations proposed by Hafkamp and colleagues include the following: (a) the frequently used IHC DO‐7 antibody binds to both normal and mutant p53 protein, (b) the *TP53* mutations occur outside the common exons 5 to 8, (c) upregulation by genotoxic insults like the aforementioned ultraviolet radiation exposure, or (d) lack of functional E6 expression.^34^, ^70^, ^76^, ^100^ For these reasons, the p53 protein was not included as a molecular parameter in the present meta‐analysis.

The present study has some limitations. First, the inclusion criterion for study selection that “the results of the molecular parameter in HNSCC of NSND had to be reported in the title or abstract” might have introduced selection bias, as the parameter could have been portrayed in the tables or full text without an explicit description of this criterion in the abstract. However, as the main aim of the present systematic review was to provide an overview of potential molecular parameters underlying head and neck carcinogenesis in NSND, reporting of important parameters in the title or abstract was assumed. Secondly, molecular parameters may have been found less potential in other studies and therefore may not have been published, resulting in publication bias. To limit this bias, articles reporting that HPV, p16 overexpression, *TP53* mutations, and p53 protein expression play no role in the head and neck carcinogenesis of NSND were included as well. Thirdly, the methodological quality assessment of the included studies showed great heterogeneity in internal and external validity across studies. Therefore, the focus during critical appraisal was on well‐described detection methods and reproducibility of the study protocol for inclusion in the meta‐analysis. Fourthly, older studies could have reported on p16 expression without knowing its correlation to HPV infection in OPSCC, therefore not applying the nowadays accepted cutoff value of ≥70% positivity or diffuse intense/strong staining in tumor tissue. As a result, possible HPV positive cases could have been excluded from the meta‐analysis, which may have an impact on the reported HPV prevalence in this study. Finally, analyses of the data on nonsmokers and nondrinkers had to be performed separately as in the majority of the studies it was unclear if these groups showed overlap in tobacco and alcohol consumption. Moreover, studies were not excluded based on their definition of the construct NSND, so consumption of either tobacco or alcohol might have played a minor role.

This systematic review summarizes the current knowledge about the underlying carcinogenic mechanisms in NSND. HNSCC in these patients is more often related to the molecular parameters HPV and tumor protein p16 overexpression compared to tumors of SD. In a third of virus‐negative tumors, *TP53* mutations were detected with a mutational profile associated with aging and ultraviolet light exposure (in lip squamous cell carcinoma) rather than to tobacco consumption. Future research should consider a strict definition of the construct nonsmoker (ie, <100 tobacco products/lifetime), whereas a less strict definition of the construct nondrinker could be opted for (ie, <1 alcoholic drink/day). For the sporadically reported molecular parameters in tumors of NSND, such as immune response and checkpoint factors including the INFγ and NFKB pathways, larger studies are needed to confirm the value of these molecular parameters in cancer diagnosis, prognosis, individualized therapy selection, or therapy effect evaluation in NSND.

## Supporting information


**Supplementary Table 1** Literature search

**Supplementary Table 2** Study quality assessment criteria

**Supplementary Table 3** Sporadically reported molecular parameters in head and neck squamous cell carcinoma of non‐smokers and non‐drinkers as presented in Figure 2

**Supplementary Table 4** REMARK based quality assessment of 57 studies reporting on HPV, p16, p53, or *TP53* mutations in head and neck squamous cell carcinoma of non‐smokers and non‐drinkers.
Abbreviations: REMARK: REporting recommendations for tumour MARKer prognostic studies^27^; HPV: human papillomavirus; NSND: non‐smokers and non‐drinkers; PCR: polymerase chain reaction; Seq: sequencing; IHC: immunohistochemistry; ISH: in situ hybridization

**Supplementary Figure 1** Sensitivity analyses evaluating the statistical reliability of the data by only retaining studies with at least ten patients in both the non‐smoking/non‐drinking and smoking/drinking groups. Since exclusion of these studies did not change the conclusions, all studies remained included in the meta‐analysis (Figure 3).

## Data Availability

The data that support the findings of this study are available from the corresponding author upon reasonable request.
