# Supplementary material for: Evidence for different molecular parameters in head and neck squamous cell carcinoma of nonsmokers and nondrinkers: Systematic review and meta‐analysis on HPV, p16, and TP53
Source: Head Neck. 2020 Oct 23;43(1):303–22. doi: 10.1002/hed.26513 (PMC7756438; doi:10.1002/hed.26513)
Supplement: Supplementary file 1 — Supplementary Table 1 Literature search [file HED-43-303-s001.docx]

**Supplementary Table 1.** Literature search

| PubMed | (“mouth”[MeSH] OR “nasopharynx”[Mesh] OR “oropharynx”[Mesh] OR “hypopharynx”[Mesh] OR “larynx”[MeSH] OR mouth[tiab] OR oral[tiab] OR pharynx[tiab] OR nasopharynx[tiab] OR oropharynx[tiab] OR hypopharynx[tiab] OR larynx[tiab] OR tongue[tiab] OR throat[tiab] OR “head and neck”[tiab]) |
| --- | --- |
|  | AND ("Neoplasms"[Mesh] OR "Carcinoma, Squamous Cell"[Mesh] OR neoplas*[tiab] OR cancer[tiab] OR malignanc*[tiab]OR tumor*[tiab] OR squamous cell carcinoma*[tiab] OR squamous carcinoma[tiab] OR epidermoid carcinoma[tiab] OR planocellular carcinoma[tiab]) |
|  | AND ("Biomarkers"[Mesh] OR "Proteins"[Mesh] OR "Immunohistochemistry"[Mesh] OR “Polymerase chain reaction”[Mesh] OR “In situ hybridization”[Mesh] OR “Sequence Analysis, DNA”[Mesh] OR "Viruses"[Mesh] OR "Genetics"[Mesh] OR Prognosis[Mesh] OR biomarker[tiab] OR ((biologic*[tiab] OR serum[tiab] OR clinical[tiab] OR viral[tiab] OR immune[tiab] OR immunologic[tiab]) AND marker[tiab]) OR genetics[tiab] OR genomics[tiab] OR immunogenetics[tiab] OR molecular biology[tiab] OR prognosis[tiab] OR prognostic factor[tiab] OR treatment outcome[tiab] OR disease-free survival[tiab] OR overall survival[tiab] OR protein[tiab] OR immunohistochemistry[tiab] OR Polymerase chain reaction[tiab] OR PCR[tiab] OR In situ hybridization[tiab] OR FISH[tiab] OR DNA Sequence Analyses[tiab] OR DNA Sequence Analysis[tiab] OR DNA Sequence Determinations[tiab] OR DNA Sequence Determination[tiab] OR DNA sequencing[tiab] OR virus[tiab] OR genomics[tiab]) |
|  | AND (non-smok*[tiab] OR never-smok*[tiab] OR non-drink*[tiab] OR never-drink* OR NSND[tiab] OR no-alcohol[tiab] OR no-tobacco[tiab]) |
| Embase | (Exp mouth/ or exp nasopharynx/ or exp oropharynx/ or exp hypopharynx/ or exp larynx/ or exp tongue/ or exp throat/ or exp "head and neck carcinoma"/ or exp "head and neck cancer"/ or exp "head and neck tumor"/ or exp "head and neck squamous cell carcinoma"/ or mouth.mp. or nasopharynx.mp. or oropharynx.mp. or hypopharynx.mp. or larynx.mp. or tongue.mp. or throat.mp.) |
|  | AND (Exp neoplasm/ or exp malignant neoplasm/ or exp squamous cell carcinoma or neoplasm.mp. or malignant neoplasm.mp. or squamous cell carcinoma.mp.) |
|  | AND (Exp biological marker/ or exp protein/ or exp cell protein/ or exp cell cycle protein/ or exp inhibitor protein/ or exp protein analysis/ or exp DNA binding protein/ or RNA binding protein/ or viral protein/ or exp immunohistochemistry/ or exp polymerase chain reaction/ or exp DNA polymerase/ or exp RNA polymerase/ or exp in situ hybridization/ or exp DNA sequence/ or exp DNA tumor virus/ or exp Epstein Barr virus/ or exp virus carcinogenesis/ or exp DNA virus infection/ or exp RNA virus/ or exp virus/ or exp cancer genetics/ or exp genetics/ or exp genomics/ or exp prognosis/ or exp cancer prognosis/ or exp cancer survival/ or exp overall survival/ or exp disease free survival or biological marker.mp. or protein.mp. or immunohistochemistry.mp. or polymerase chain reaction.mp. or in situ hybridization.mp. or DNA sequence.mp. or virus.mp. or genetics.mp. or genomics.mp. or prognosis.mp. or overall survival.mp. or disease free survival.mp.) |
|  | AND (non-smoker.mp. or non-smoking.mp. or never-smoker.mp. or never-smoking.mp. or non-drinker.mp. or non-drinking.mp. or never-drinker.mp. or never-drinking.mp. or no-tobacco.mp. or no-alcohol.mp.) |
| Google Scholar* | allintitle: (oral OR oropharyngeal OR laryngeal OR tongue OR "head and neck") |
|  | AND (neoplasm OR carcinoma OR cancer OR tumor) |
|  | AND (marker OR "non smoking” OR “non drinking” OR “no tobacco” OR “no alcohol”) |

* Not including citations or patents
