# Supplementary material for: Evidence for different molecular parameters in head and neck squamous cell carcinoma of nonsmokers and nondrinkers: Systematic review and meta‐analysis on HPV, p16, and TP53
Source: Head Neck. 2020 Oct 23;43(1):303–22. doi: 10.1002/hed.26513 (PMC7756438; doi:10.1002/hed.26513)
Supplement: Supplementary file 2 — Supplementary Table 2 Study quality assessment criteria [file HED-43-303-s002.docx]

**Supplementary Table 2.** Study quality assessment criteria

| *1. Was an explicit definition of non-smokers and/or non-drinkers provided?* | | |
| --- | --- | --- |
|  | Yes | Clear definition including cut-off values |
|  | Unclear | General definition |
|  | No | No definition |
| *2. Was the sample of patients representative of the patients who will receive the biomarker test in clinical practice?* | | |
|  | Yes | All consecutive patients in a defined time period are included |
|  | Unclear | Selection not described or unclear if patients were consecutively included |
|  | No | Patients were selected |
| *3. Were the patient selection criteria clearly described?* | | |
|  | Yes | Both inclusion and exclusion criteria described |
|  | Unclear | Only inclusion criteria described |
|  | No | No inclusion or exclusion criteria described |
| *4. Were the specimen characteristics clearly described?* | | |
|  | Yes | Biological material, pTNM, and method of preservation described |
|  | Unclear | Only two of the abovementioned criteria described |
|  | No | Less than two criteria described |
| *5. Were the assay methods clearly described in sufficient detail to permit replication of the test(s)? More specific, was a description provided of the assay method, detailed protocol, specific reagents or kits used, quality control procedures, reproducibility assessments, quantification methods, and scoring and reporting protocols?* | | |
|  | Yes | IHC: description of antigen retrieval, clone, scoring, staining platform, and endogenous peroxidase/alcalic phosphatase blocking |
|  |  | PCR: description of DNA/RNA isolation, primers, controls (including humane control of DNA quality), PCR conditions, and confirmation of PCR product |
|  |  | ISH: description of probe or kit, evaluation criteria, pre-treatment, detection method, and controls |
|  |  | Sequencing: description of DNA/RNA isolation, virus specific controls, scoring, PCR conditions, sequencing method and analysis |
|  | Unclear | IHC: description of antigen retrieval, clone, and scoring |
|  |  | PCR: description of DNA/RNA isolation and primers |
|  |  | ISH: description of probe or kit and evaluation criteria |
|  |  | Sequencing: description of DNA/RNA isolation, virus specific controls, and scoring |
|  | No | IHC: no description of antigen retrieval, clone, or scoring |
|  |  | PCR: no description of DNA/RNA isolation or primers |
|  |  | ISH: no description of probe/ kit or evaluation criteria |
|  |  | Sequencing: no description of DNA/RNA isolation, virus specific controls, or scoring |
| *6. Did all patients receive the same biomarker test(s)?* | | |
|  | Yes | All tests were done on all included patients |
|  | Unclear | Specific test on subgroup including justification for subgroup |
|  | No | Specific test on subgroup without explicit justification |
| *7. Were the biomarker tests interpreted without knowledge of the condition of the patient?* | | |
|  | Yes | Explicit statement the test interpretation was done without knowledge of clinical parameters or the test was performed during routine diagnostics |
|  | Unclear | No remarks regarding blinded test interpretation |
|  | No | Clinical parameters, including smoking and drinking status, were known to test interpreters |
| *8. Were test-retest (reproducibility) statistics applied?* | | |
|  | Yes | Biomarker confirmed by two or more techniques or data interpretation done by two independent raters |
|  | Unclear | Data interpretation not described |
|  | No | One technique used or one rater interpreting the data |
| *9. Were appropriate statistical methods applied?* | | |
|  | Yes | Both univariate and multivariable analysis of biomarker in NSND, including rationale for statistical model building |
|  | Unclear | Only univariate analysis of biomarker in NSND |
|  | No | No specific statistics for NSND |
| *10. Was the applied biomarker clinically relevant or applicable in this patient population?* | | |
|  | Yes | HPV: confirmation of HPV with two techniques (p16 only valid as a technique in OPSCC) |
|  |  | p16: ≥70% positivity or diffuse intense/ strong staining in tumor tissue |
|  |  | p53: too much heterogeneity for a clear cut-off value |
|  |  | *TP53*: mutations in at least exon 5-8 |
|  | Unclear | If it is not 'yes' |
|  | No | The only four molecular parameters left are all potentially clinically relevant. |
