# Supplementary material for: Evidence for different molecular parameters in head and neck squamous cell carcinoma of nonsmokers and nondrinkers: Systematic review and meta‐analysis on HPV, p16, and TP53
Source: Head Neck. 2020 Oct 23;43(1):303–22. doi: 10.1002/hed.26513 (PMC7756438; doi:10.1002/hed.26513)
Supplement: Supplementary file 3 — Supplementary Table 3 Sporadically reported molecular parameters in head and neck squamous cell carcinoma of non‐smokers and non‐drinkers as presented in Figure 2 [file HED-43-303-s003.docx]

**Supplementary Table 3.** Sporadically reported molecular parameters in head and neck squamous cell carcinoma of non-smokers and non-drinkers as presented in Figure 2

| **Molecular parameter** | **First author** | **Year** |
| --- | --- | --- |
| *PIK3CA* | Li | 2015 |
|  | Pickering | 2014 |
|  | Tan | 2014 |
| Cyclin D1 | Haas | 2002 |
|  | Wangsa | 2016 |
| DDX3 | Heerma van Voss | 2015 |
|  | Lee | 2014 |
| EGFR | Ryott | 2009 |
|  | Wangsa | 2016 |
| *FAT1* | Li | 2015 |
|  | Pickering | 2014 |
| INFγ pathway | Farshadpour | 2012 |
|  | Foy | 2017 |
| *NOTCH1* | Li | 2015 |
|  | Upadyay | 2016 |
| *CDKN2a* | Heaton | 2014 |
| C-myc | Perez-Sayans | 2014 |
| DNMT1 | Daniel | 2010 |
| GTSP1 | Soares | 2017 |
| IDO-1 | Foy | 2017 |
| IL-10 | Chuang | 2012 |
| MSI | Field | 1995 |
| NFKB pathway | Farshadpour | 2012 |
| PD-L1/PD-1 | Foy | 2017 |
| pRb | Haas | 2002 |
| *PRR4* | Ekizoglu | 2018 |
| Prx1 | Niu | 2016 |
| *SFRP4* | Marsit | 2006 |
| SLPI | Quabius | 2015 |
| TILs | Foy | 2017 |
| *TWIST2* | Zhu | 2017 |

**References**

Chuang CY, Sung WW, Wang L, et al. Differential impact of IL-10 expression on survival and relapse between HPV16-positive and -negative oral squamous cell carcinomas. PLoS One. 2012;7:e47541.

Daniel FI, Rivero ER, Modolo F, Lopes TG, Salum FG. Immunohistochemical expression of DNA methyltransferases 1, 3a and 3b in oral leukoplakias and squamous cell carcinomas. Arch Oral Biol. 2010;55:1024-1030.

Ekizoglu S, Ulutin T, Guliyev J, Buyru N. PRR4: A novel downregulated gene in laryngeal cancer. Oncology Letters. 2018;15:4669-4675.

Farshadpour F, Hordijk G, Koole R, Slootweg P. Head and neck squamous cell carcinoma in non‐smoking and non‐drinking patients with multiple tumors: etiologic significance of p53 and Ki‐67 in non‐tumorous epithelium. Journal of oral pathology & medicine. 2008;37:549-554.

Field JK, Kiaris H, Howard P, Vaughan ED, Spandidos DA, Jones AS. Microsatellite instability in squamous cell carcinoma of the head and neck. Br J Cancer. 1995;71:1065-1069.

Foy JP, Bertolus C, Michallet MC, et al. The immune microenvironment of HPV-negative oral squamous cell carcinoma from never-smokers and never-drinkers patients suggests higher clinical benefit of IDO1 and PD1/PD-L1 blockade. Ann Oncol. 2017;28:1934-1941.

Haas S, Hormann K, Bosch FX. Expression of cell cycle proteins in head and neck cancer correlates with tumor site rather than tobacco use. Oral Oncology. 2002;38:618-623.

Heerma van Voss MR, van Kempen PMW, Noorlag R, van Diest PJ, Willems SM, Raman V. DDX3 has divergent roles in head and neck squamous cell carcinomas in smoking versus non-smoking patients. Oral Diseases. 2015;21:270-271.

Heaton CM, Durr ML, Tetsu O, Van Zante A, Wang SJ. TP53 and CDKN2a mutations in never-smoker oral tongue squamous cell carcinoma. Laryngoscope. 2014;124:E267-E273.

Lee CH, Lin SH, Yang SF, et al. Low/negative expression of DDX3 might predict poor prognosis in non-smoker patients with oral cancer. Oral Dis. 2014;20:76-83.

Li R, Faden DL, Fakhry C, et al. Clinical, genomic, and metagenomic characterization of oral tongue squamous cell carcinoma in patients who do not smoke. Head Neck. 2015;37:1642-1649.

Marsit CJ, McClean MD, Furniss CS, Kelsey KT. Epigenetic inactivation of the SFRP genes is associated with drinking, smoking and HPV in head and neck squamous cell carcinoma. Int J Cancer. 2006;119:1761-1766.

Niu W, Zhang M, Chen H, et al. Peroxiredoxin 1 promotes invasion and migration by regulating epithelial-to-mesenchymal transition during oral carcinogenesis. Oncotarget. 2016;7:47042-47051.

Perez-Sayans M, Suarez-Penaranda JM, Padin-Iruegas E, et al. Quantitative determination of c-myc facilitates the assessment of prognosis of OSCC patients. Oncology Reports. 2014;31:1677-1682.

Pickering CR, Zhang J, Neskey DM, et al. Squamous cell carcinoma of the oral tongue in young non-smokers is genomically similar to tumors in older smokers. Clin Cancer Res. 2014;20:3842-3848.

Quabius ES, Gorogh T, Fischer GS, et al. The antileukoprotease secretory leukocyte protease inhibitor (SLPI) and its role in the prevention of HPV-infections in head and neck squamous cell carcinoma. Cancer Lett. 2015;357:339-345.

Ryott M, Wangsa D, Heselmeyer-Haddad K, et al. EGFR protein overexpression and gene copy number increases in oral tongue squamous cell carcinoma. Eur J Cancer. 2009;45:1700-1708.

Soares PDO, Cury PM, Lopez RVM, et al. GTSP1 expression in non-smoker and nondrinker patients with squamous cell carcinoma of the head and neck. PLoS ONE. 2017;12 (8) (no pagination):

Tan DS, Wang W, Leong HS, et al. Tongue carcinoma infrequently harbor common actionable genetic alterations. BMC Cancer. 2014;14:679.

Upadhyay P, Nair S, Kaur E, et al. Notch pathway activation is essential for maintenance of stem-like cells in early tongue cancer. Oncotarget. 2016;7:50437-50449.

Wangsa D, Chowdhury SA, Ryott M, et al. Phylogenetic analysis of multiple FISH markers in oral tongue squamous cell carcinoma suggests that a diverse distribution of copy number changes is associated with poor prognosis. International Journal of Cancer. 2016;138:98-109.

Zhu Y, Zhang W, Wang P. Smoking and gender modify the effect of TWIST on patient survival in head and neck squamous carcinoma. Oncotarget. 2017;8:85816-85827.
