# Supplementary material for: Evidence for different molecular parameters in head and neck squamous cell carcinoma of nonsmokers and nondrinkers: Systematic review and meta‐analysis on HPV, p16, and TP53
Source: Head Neck. 2020 Oct 23;43(1):303–22. doi: 10.1002/hed.26513 (PMC7756438; doi:10.1002/hed.26513)
Supplement: Supplementary file 4 — Supplementary Table 4 REMARK based quality assessment of 57 studies reporting on HPV, p16, p53, or TP53 mutations in head and neck squamous cell carcinoma of non‐smokers and non‐drinkers. Abbreviations: REMARK: REporting recommendations for tumour MARKer prognostic studies27; HPV: human papillomavirus; NSND: non‐smokers and non‐drinkers; PCR: polymerase chain reaction; Seq: sequencing; IHC: immunohistochemistry; ISH: in situ hybridization [file HED-43-303-s004.docx]

**Supplementary Table 4.** REMARK based quality assessment of 57 studies reporting on HPV, p16, p53, or *TP53* mutations in head and neck squamous cell carcinoma of non-smokers and non-drinkers.

| Author | 1. Definition NSND | 2. Representative patients | 3. Patient selection criteria | 4. Specimen characteristics | 5. Clear assay method | 6. All patients same test | 7. Blinded marker interpretation | 8. Test-retest applied | 9. Appropriate statistics | 10. Clinically relevant |
| --- | --- | --- | --- | --- | --- | --- | --- | --- | --- | --- |
| Amsbaugh et al.^78^ | Unclear | Yes | Yes | Unclear | No | No | Yes | Unclear | No | Unclear |
| Andrews et al.^17^ | Unclear | Yes | Yes | Unclear | Unclear | Yes | Unclear | Yes | Yes | Yes |
| Ang et al.^79^ | Unclear | Yes | Yes | Yes | Unclear | Yes | Unclear | Unclear | Yes | Unclear |
| Angiero et al.^52^ | Unclear | Unclear | Unclear | Unclear | HPV yes  p16, p53 no | Unclear | Unclear | HPV yes  p16, p53 no | Unclear | HPV yes  p16, p53 unclear |
| Antonsson et al.^43^ | Unclear | Yes | Unclear | Yes | Yes | Yes | Yes | HPV yes  p16 no | HPV yes  p16 no | HPV yes  p16 |
| Bragelmann et al.^53^ | Yes | No | Unclear | Yes | No | Yes | Unclear | Yes | Not applicable | Yes |
| Chen et al.^32^ | Yes | Yes | Yes | Unclear | Unclear | Yes | Unclear | Unclear | Yes | Unclear |
| Chen et al.^80^ | Unclear | Unclear | Unclear | Yes | Unclear | Yes | Unclear | Unclear | Unclear | Yes |
| Chen et al.^81^ | Unclear | Yes | Yes | Unclear | Yes | Yes | Yes | Yes | Unclear | Unclear |
| Chuang et al.^82^ | Unclear | Unclear | Unclear | Yes | HPV unclear | Yes | Unclear | Unclear | Yes | Unclear |
| Dediol et al.^33^ | Yes | Yes | Yes | Yes | HPV unclear  p16 no | Yes | Unclear | HPV unclear p16 no | Yes | Unclear |
| Descamps et al.^83^ | Unclear | No | Unclear | Yes | Unclear | Unclear | Unclear | Yes | Yes | Yes |
| Faden et al.^99^ | Unclear | No | Unclear | Unclear | Yes | Yes | No | Unclear | No | Unclear |
| Farnebo et al.^84^ | Unclear | Unclear | Unclear | Unclear | HPV unclear  *TP53* no | Yes | Unclear | No | Unclear | Unclear |
| Farshadpour et al.^18^ | Unclear | Yes | Unclear | Yes | Yes | Yes | Yes | Yes | Unclear | Yes |
| Fernandez-Acenero et al.^54^ | Unclear | Unclear | Unclear | Unclear | Unclear | Yes | No | Unclear | Unclear | Unclear |
| Field et al.^29^ | Yes | No | Unclear | Unclear | Yes | Yes | Unclear | Yes | Unclear | Unclear |
| Fouret et al.^34^ | Yes | Yes | Yes | Yes | Yes | Yes | Unclear | Unclear | Unclear | Unclear |
| Gillison et al.^35^ | Yes | Yes | Yes | Unclear | Yes | Yes | Unclear | Unclear | Yes | Unclear |
| Gillison et al.^95^ | Unclear | Yes | Yes | Yes | Unclear | Yes | Unclear | Unclear | Yes | Yes |
| Gonzalez-Ramirez et al.^85^ | Unclear | Unclear | Yes | Yes | PCR unclear  Seq yes | Yes | Unclear | Unclear | Unclear | Yes |
| Haas et al.^59^ | Unclear | No | Unclear | Unclear | Yes | Yes | Unclear | Unclear | Unclear | Unclear |
| Habbous et al.^4^ | Yes | Yes | Unclear | Unclear | No | No | Yes | No | Unclear | Unclear |
| Hafkamp et al.^86^ | Yes | Unclear | Unclear | Yes | Yes | No | Unclear | Yes | Yes | Unclear |
| Heaton et al.^41^ | Yes | Yes | Yes | Yes | Yes | Yes | Unclear | Unclear | Yes | Yes |
| Hess et al.^46^ | Unclear | Yes | Unclear | Unclear | No | Yes | Yes | Unclear | Unclear | Yes |
| Hoffmann et al.^87^ | Yes | Unclear | Unclear | Yes | IHC, Seq no  PCR unclear | Yes | Unclear | Yes | Yes | Unclear |
| Hong et al.^67^ | Unclear | Yes | Yes | Yes | p16 no  HPV unclear | Yes | Unclear | Yes | Unclear | Yes |
| Hong et al.^49^ | Unclear | Unclear | Unclear | Yes | Yes | Yes | Unclear | Yes | Unclear | Yes |
| Joo et al.^88^ | Unclear | Unclear | Yes | Yes | Unclear | Yes | Unclear | No | Yes | Unclear |
| Kalfert et al.^96^ | Unclear | Unclear | Unclear | Yes | Yes | Yes | Unclear | Unclear | Unclear | Unclear |
| Karpathiou et al.^60^ | Unclear | Unclear | Yes | Yes | No | Yes | Unclear | Yes | Unclear | p16 yes  p53 unclear |
| Laco et al.^19^ | Unclear | Yes | Yes | Yes | Yes | Yes | Unclear | Yes | No | Unclear |
| Li et al.^89^ | Unclear | No | Yes | Yes | Yes | Yes | Unclear | Unclear | No | Yes |
| Mafune et al.^42^ | Yes | Yes | Yes | Unclear | p16 no  *TP53* yes | Yes | Unclear | Unclear | Yes | Yes |
| Maruyama et al.^58^ | Yes | Yes | Unclear | Yes | Yes | No | Unclear | Unclear | Yes | Yes |
| Matthews et al.^61^ | Yes | Unclear | Yes | Unclear | Yes | Yes | Yes | No | Unclear | Unclear |
| Mena et al.^44^ | Unclear | Yes | Yes | Unclear | p16 no  PCR unclear | Yes | Yes | Yes | Yes | Yes |
| Mirghani et al.^50^ | Unclear | No | Unclear | Yes | p16, *TP53* yes  PCR unclear | Yes | Unclear | Yes | No | Yes |
| Oliveira et al.^31^ | Unclear | Unclear | Yes | Unclear | Yes | Yes | Unclear | Unclear | Unclear | Unclear |
| Ostwald et al.^62^ | Unclear | No | Unclear | Yes | Unclear | Yes | Unclear | Yes | Unclear | Yes |
| Peterson et al.^36^ | Yes | Yes | Yes | Unclear | Unclear | No | Unclear | Unclear | Yes | Unclear |
| Pickering et al.^100^ | Yes | No | Unclear | Yes | Yes | Yes | Unclear | Unclear | Unclear | Unclear |
| Platek et al.^90^ | Unclear | Yes | Yes | Unclear | No | Yes | Yes | Unclear | Yes | Unclear |
| Poling et al.^91^ | Yes | No | Unclear | Yes | p16 unclear  ISH yes | Yes | Unclear | Unclear | No | Yes |
| Quabius et al.^92^ | Unclear | Unclear | Unclear | Unclear | PCR unclear  Seq no | Yes | Unclear | Yes | Unclear | Yes |
| Ralli et al.^97^ | No | Unclear | Yes | Unclear | No | Yes | Unclear | Unclear | Unclear | Yes |
| Schlecht et al.^45^ | Unclear | No | Unclear | Yes | PCR unclear  Seq yes | Yes | Unclear | Yes | Unclear | Yes |
| Siebers et al.^37^ | Yes | Yes | Yes | Yes | Yes | Yes | No | Yes | Not applicable | Yes |
| Silva et al.^47^ | Unclear | Unclear | Unclear | Yes | Yes | Yes | Yes | Yes | Unclear | Unclear |
| Simonato et al.^93^ | Unclear | Unclear | Unclear | Unclear | Yes | Yes | Unclear | Unclear | Unclear | Unclear |
| Tachezy et al.^38^ | Yes | Yes | Yes | Unclear | Yes | Yes | Unclear | Yes | Unclear | Yes |
| Tan et al.^101^ | Unclear | Yes | Unclear | Yes | Yes | Yes | Unclear | Unclear | Unclear | Unclear |
| Tsimplaki et al.^30^ | Unclear | Yes | Unclear | Unclear | Unclear | Yes | Unclear | Yes | Unclear | Yes |
| Vatca et al.^39^ | Yes | Yes | Yes | Yes | Unclear | No | Yes | Unclear | Yes | Unclear |
| Wangsa et al.^94^ | Unclear | Unclear | Unclear | Unclear | HPV no  *TP53* unclear | No | Unclear | Unclear | Yes | Unclear |
| Xu et al.^40^ | Yes | Yes | Yes | Yes | p16 no  HPV unclear | Yes | Unclear | Yes | Unclear | Yes |
| Ye et al.^98^ | Unclear | Yes | Yes | Yes | No | Yes | Unclear | Unclear | Unclear | Unclear |
| Zanaruddin et al.^51^ | Unclear | No | Yes | Yes | Yes | Yes | Unclear | Yes | Unclear | Yes |
| Zhao et al.^48^ | Unclear | Yes | Yes | Yes | Unclear | Yes | Yes | Yes | Yes | p16 yes  HPV unclear |

Abbreviations: REMARK: REporting recommendations for tumour MARKer prognostic studies^27^; HPV: human papillomavirus; NSND: non-smokers and non-drinkers; PCR: polymerase chain reaction; Seq: sequencing; IHC: immunohistochemistry; ISH: in situ hybridization
