# Supplementary material for: Evidence for different molecular parameters in head and neck squamous cell carcinoma of nonsmokers and nondrinkers: Systematic review and meta‐analysis on HPV, p16, and TP53
Source: Head Neck. 2020 Oct 23;43(1):303–22. doi: 10.1002/hed.26513 (PMC7756438; doi:10.1002/hed.26513)
Supplement: Supplementary file 5 — Supplementary Figure 1 Sensitivity analyses evaluating the statistical reliability of the data by only retaining studies with at least ten patients in both the non‐smoking/non‐drinking and smoking/drinking groups. Since exclusion of these studies did not change the conclusions, all studies remained included in the meta‐analysis (Figure 3). [file HED-43-303-s005.docx]

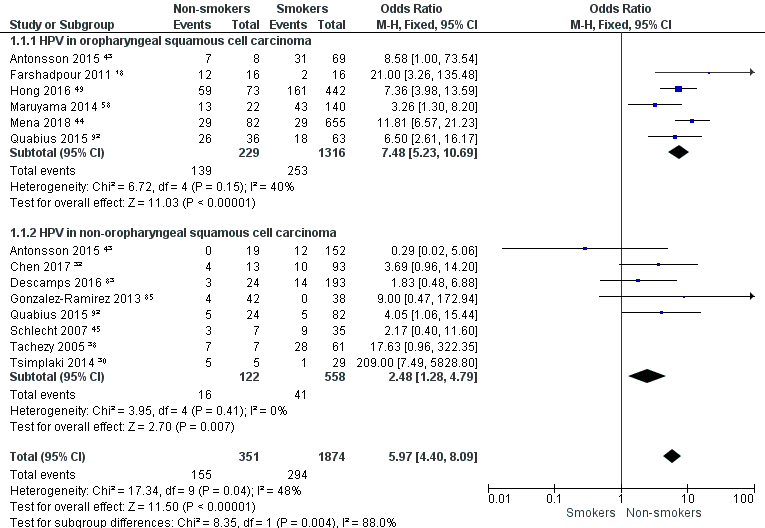


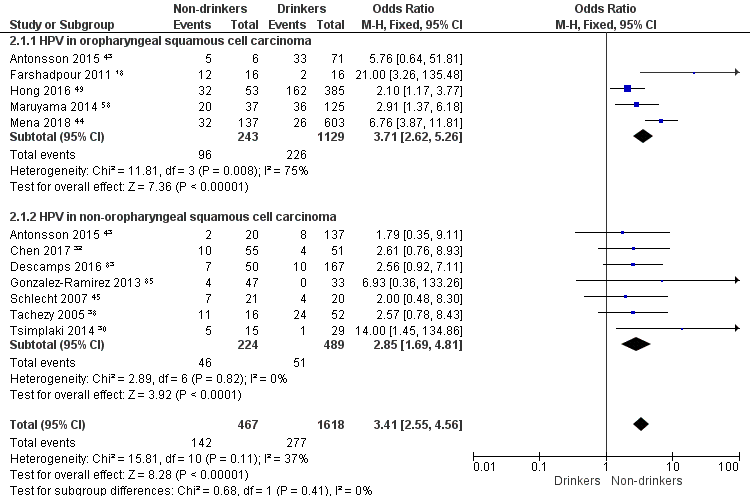


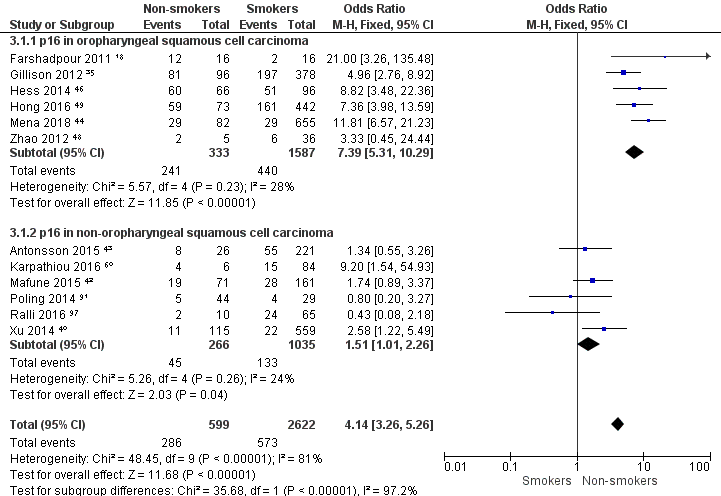


**Supplementary Figure 1.** Sensitivity analyses evaluating the statistical reliability of the data by only retaining studies with at least ten patients in both the non-smoking/non-drinking and smoking/drinking groups. Since exclusion of these studies did not change the conclusions, all studies remained included in the meta-analysis (Figure 3).
